# From Ecological Threat to Bioactive Resource: The Nutraceutical Components of Blue Crab (*Callinectes sapidus*)

**DOI:** 10.3390/ijms27010381

**Published:** 2025-12-30

**Authors:** Annalaura Brai, Lorenzo Tiberio, Matteo Chiti, Federica Poggialini, Chiara Vagaggini, Guia Consales, Letizia Marsili, Elena Dreassi

**Affiliations:** 1 Department of Biotechnology, Chemistry and Pharmacy, University of Siena, Via A. Moro, 53100 Siena, Italy; annalaura.brai@unisi.it (A.B.); lorenzo.tiberio@student.unisi.it (L.T.); federic.poggialini@unisi.it (F.P.); chiara.vagaggini@student.unisi.it (C.V.); 2 Department of Physical Science, Earth and Environment, Università degli Studi di Siena, 4, Via P.A. Mattioli, 53100 Siena, Italy; m.chiti7@student.unisi.it (M.C.); guia.consales@unisi.it (G.C.); letizia.marsili@unisi.it (L.M.)

**Keywords:** alien species, type-2 diabetes, antioxidants, astaxanthin, angiotensin-converting enzyme

## Abstract

Native to the western Atlantic, the Atlantic blue crab *Callinectes sapidus* (CS) has spread to the Mediterranean, affecting local ecosystems and mussel aquaculture and leading to severe ecological and financial losses in Italy and other areas. Given its rapid spread and socio-economic impacts, several countries have begun to exploit CS commercially, but the consumers’ interest is still limited. In this study, we analysed both nutrient and nutraceutical profiles of CS meats, evaluating potential differences related to sex and meat typology. We found that CS meats are rich in high quality proteins and contain all the essential amino acids required for a correct diet. The fat of CS is not only rich in polyunsaturated fatty acids (PUFAs) but also displays remarkably low atherogenicity and thrombogenicity indices, highlighting its strong potential in promoting cardiovascular health. In addition, CS is rich in nutraceutical compounds, in particular polyphenols and astaxanthin, revealing a good antioxidant activity maintained after simulated gastrointestinal hydrolysis. Last but not least, CS has remarkable α-glucosidase and angiotensin-converting enzyme inhibitory activity, highlighting potential benefits in controlling glycaemic peaks and hypertension.

## 1. Introduction

According to Article 3 of Regulation (EU) No. 1143/2014, an “alien species” is any animal, plant, fungus, or microorganism introduced outside its natural range. Alien species are defined as invasive alien species (IAS) when they negatively impact biodiversity, ecosystems, or the economy [1]. IAS are closely linked to economic activity, with trade being a major driver of their spread; interestingly, the volume of international commerce can serve as a useful predictor of the number of IAS in a given country [2,3]. Maritime transport, through ballast water and organisms attached to ship hulls, is one of the most common and almost inevitable mechanisms for the spread of aquatic alien species, largely driven by globalised shipping and trade.

In this regard, the blue crab *Callinectes sapidus* (CS), which is endemic to the western Atlantic, has spread throughout areas of the Mediterranean and is regarded as invasive [4,5]. The Mediterranean Sea shows significant regional ecological variability, and while some studies have been conducted in parts of this basin, there is still a lack of data on blue crabs caught near the Italian coast [6]. Although the invasion of the Mediterranean by CS has become increasingly evident only in recent years, the species is not yet listed in the Global Invasive Species Database as of November 2025 [7]. In the meantime, several Member States, most in particular Italy, have developed national and regional programs to manage and contain this species, particularly in regions where it has an impact on nearby ecosystems and fisheries. FAO data on its introduction in Italy remain limited, but negative economic impacts are already recognised [8,9]. In 2023, an unprecedented surge of CS in the northern Adriatic caused significant ecological and economic damage. The mussel aquaculture has been threatened by the spread of the blue crab, especially along the Adriatic coast and coastal lagoons. Manila clam production fell by 71.8% (≈ EUR 65 million annually) in the Sacca di Goro (FE, Italy), and small-scale fisheries also experienced losses [10]. The Italian government acknowledges the threat posed by CS (the Atlantic blue crab) and *Portunus segnis* (the African blue swimmer crab) through Ministerial Decree No. 587931, which allocates funding to aquaculture consortia and fisheries for the capture and disposal of these invasive crabs [11]. As a generalist predator, CS mainly eats bivalves, like mussels, which reduces harvest yields and disturbs local ecosystems [12,13,14]. Although trapping and monitoring can be helpful, coordinated management techniques are required to safeguard mussel aquaculture from this invasive species [14]. Specifically, CS are voracious, reproduce rapidly, and can damage fishing gear by entangling and cutting nets, further contributing to economic losses [15,16]. Early studies are therefore crucial to address the threat and potentially transform the invasive species into a valuable resource [17]. Data on the impacts of alien species remain incomplete due to the complexity and unpredictability of their ecological and socio-economic effects, as well as the lack of systematic monitoring. Even if the economic impacts are evident in countries experiencing its invasion, other nations have successfully commercialised the CS as a food. The most effective strategy against invasive alien species is prevention, since eradication is costly, difficult, and often impossible. This has driven increased research in recent years on the CS, to transform it from a harmful invasive species into a potential resource. Interestingly, the CS is abundant in the Gulf of Mexico and U.S. Atlantic coast, where it is considered an important food and socio-economic resource to the point that in some areas overfishing has even occurred [18,19,20]. Accordingly, some countries, including Egypt, Greece, Lebanon, Syria, Turkey, and Tunisia, have learned to exploit this crustacean commercially [21,22].

Herein, with the purpose of understanding the potential benefits of CS as a food, we focused our study on the evaluation of both the nutritional and the nutraceutical properties useful for human health. Although the CS has been increasingly studied in the Mediterranean, most research to date has focused on its ecology, population dynamics, and trophic impacts, or on the proximate composition of tissues from regions such as the Adriatic Sea and the eastern Mediterranean [17,23,24,25]. Nutritional investigations have documented high-quality protein, essential amino acids, and favorable fatty acid profiles in muscle and appendage tissues, as well as mineral content relevant for human nutrition [4,26]. However, nutraceutical studies have predominantly targeted processing by-products, including shells, residual chelae, and hepatopancreas, from which bioactive compounds such as chitosan, astaxanthin, and protein hydrolysates have been extracted and tested for in vitro antioxidant or cytoprotective activity [17,25,27]. Critically, the bioactive potential of edible tissues themselves, particularly under conditions simulating human gastrointestinal digestion, remains largely unexplored. Moreover, there are currently no published data on the nutritional, chemical, or functional properties of CS populations from the Tuscan coast of Italy, representing a clear geographic gap in the literature.The present study addresses these limitations by providing a comprehensive and integrated evaluation of the nutritional and chemical profile of CS collected along the Tuscan coast, with emphasis on compounds and activities of relevance to human health. Specifically, the study quantifies astaxanthin in edible tissues, assesses antioxidant activity following simulated gastrointestinal digestion, and evaluates inhibitory effects on angiotensin-converting enzyme (ACE) and α-glucosidase, linking chemical composition to potential nutraceutical functionality. By focusing on edible tissues rather than by-products and on an understudied geographic area, this work provides novel insights into the valorisation of CS as both a nutritional and functional food resource, bridging gaps between food chemistry, nutraceutical research, and invasive species management.

## 2. Results and Discussion

### 2.1. Determination of Proximate Composition

*Callinectes sapidus* (CS) exemplars were collected from the Ombrone River in September 2023 (Grosseto, Italy). Fifteen male (M) and fifteen female (F) specimens were selected, and the tissues were separated into breast (B) and claw (C) meat (MB, MC, FB, FC). The meats were then analysed for their proximate composition. As reported in Table 1, water content and proteins show no significant differences among the groups (*p* > 0.05).

Water content in the four groups comprises between 76.47 and 77.3%, with no differences related to sex or meat typology. The value is comparable to the data already reported for crustaceans, in general, comprising between 75–80% of fresh weight [24,26,28]. Previous data reported for CS highlighted a water content comprising between 66.4 and 86.7% for body meat and 65.6 to 83.1% for claw meats, reflecting seasonal, reproductive, and geographical variability. According to data already reported, the fat content is very low, accounting for 0.47 to 0.65% of fresh weight, and it is significantly higher in breast tissues, with no sex-related differences [26,28,29]. While these values are broadly comparable to other crustacean species such as shrimps and prawns and slightly below those reported for lobsters, it is important to consider that fat content in crustaceans can vary depending on season, reproductive stage, and diet, making direct comparisons between species indicative rather than definitive. The protein content is high, accounting for 18–19% of FW with no significant differences related to sex or meat typology (*p* > 0.05) [4,24,26].

Bioaccessibility of nutrients and nutraceuticals is fundamental to correctly evaluate the benefits of foods. Accordingly, gastrointestinal digestion (GID) of CS meats was simulated in vitro by sequential enzymatic treatment. First, the CS bolus was incubated with pepsin at pH 2 for 2 h to simulate gastric digestion (GD). Then the hydrolysed sample was incubated with trypsin and chymotrypsin at pH 6.5 to mimic duodenal conditions. The digestibility values, based on the initial weight, ranged from 92.11 to 92.79%, confirming high digestibility of the CS as for other crustaceans [30]. Even in this case, no differences were found between the groups analysed. The ^1^HNMR analysis of the digested samples revealed a prevalence of amino-acid-based compounds, including alanine, asparagine, aspartate, glutamate, glutamine, glycine, isoleucine, leucine, lysine, methionine, phenylalanine, taurine, tryptophan, tyrosine, and valine. Other secondary metabolites included lactic acid, trigonelline, glucose, and betaine. Signals of ATP and uracil were also detected.

The hydrolysed samples were then subjected to different analyses. To determine the content of essential amino acids, samples were subjected to column pre-derivatisation and analysed by HPLC-MS. As reported in Table 2, the total content of essential amino acids (EAA) shows no statistically significant differences in the groups *p* < 0.05. The content of individual amino acids shows no significant differences, indicating a common profile among the groups, although Thr and Val show some moderate variation. Thr shows moderate variability, with levels in MC lower than those in the other three groups, which do not differ significantly from each other (*p* < 0.05). Val also showed differences, with MB and MC grouped together and FB and FC forming a distinct cluster. According to the FAO/WHO guidelines, a 200 g portion of CS meat is sufficient to meet the complete daily requirement of essential amino acids for an adult weighing 70 kg [31]. Notably, Phe was identified as the limiting amino acid, but all other essential amino acids are present in adequate amounts. This highlights the high nutritional quality of blue crab proteins, which provide a balanced amino acid profile suitable for human dietary requirements. It should be noted that the analysis was performed after simulated gastrointestinal digestion, so dipeptides and tripeptides were not quantified; however, the data represent the amino acids available for absorption after digestion.

### 2.2. Analysis of the Lipidic Composition

**Figure 1:**
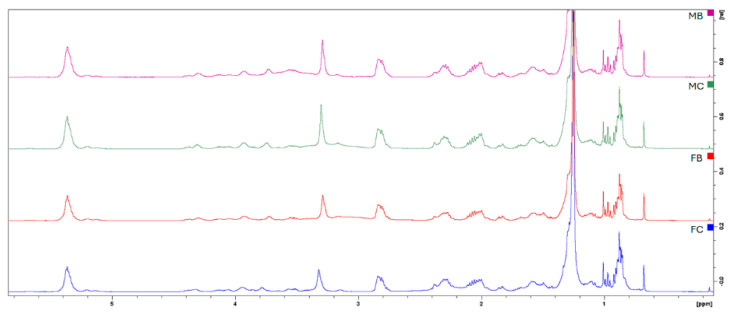
^1^HNMR of the lipophilic extracts. Superimposition of male body meat (MB, violet), male claw meat (MC, green), female body meat (FB, red), female claw meat (FC, blue).

The lipophilic fat extracts were used to determine the fat composition by ^1^HNMR and GC analyses. As reported in Figure 1, the ^1^HNMR of lipidic extracts shows the signals of saturated and unsaturated fatty acids, cholesterol, and phospholipids. Methyl groups of cholesterol and fatty acids appear at 0.68–1.00 ppm, with signals at 0.68, 0.92, and 1.00 ppm (C26, C25, C24). In the same range, the signals of saturated and monounsaturated fatty acids (SFAs and MUFAs) are visible at 0.89 ppm, and at 0.97 ppm, the ω3 polyunsaturated fatty acids (PUFAs) are visible. The methylene protons of fatty acid chains resonate between 1.18 and 1.39 ppm. The β-methylene protons adjacent to carboxyl groups appear at 1.44–1.64 ppm for most fatty acids and at 1.64–1.74 ppm for EPA (20:5 ω3). Allylic protons in MUFAs and PUFAs are observed at 1.92–2.06 ppm, with allylic protons of ω3 PUFAs at 2.05–2.13 ppm. α-Methylene protons adjacent to the carboxyl group resonate at 2.20–2.37 ppm for most fatty acids, while DHA (22:6 ω3) exhibits a distinct signal at 2.38 ppm. Polyunsaturated fatty acids display additional resonances at 2.74–2.89 ppm corresponding to conjugated methylene groups. Phosphatidylcholine signals include the trimethylammonium group at 3.32 ppm, CH_2_N at 3.78 ppm, and CH_2_OP at 4.33 ppm. Glycerol protons resonate at 3.88–4.01 ppm, while the sn2 proton appears at 5.22 ppm. Finally, olefinic protons of MUFAs and PUFAs are observed between 5.26 and 5.45 ppm.

To further characterise the detected metabolites, GC-MS analysis was carried out on the transesterified lipid extracts, and the fatty acids were subsequently quantified using gas chromatography with a flame ionisation detector (GC-FID).

As reported in Table 3, the total content of saturated fatty acids (SFA) is comparable among MB, MC, and FC (33.720–35.234%), while FB shows a higher level (38.703%). Palmitic acid (C16:0) is the most abundant SFA (15.602–17.444%), followed by stearic acid (C18:0, 10.520–14.182%). Monounsaturated fatty acids (MUFA) are higher in males (20.844% in breast, 21.492% in claw) than in females (18.045% and 17.403%, respectively), with oleic acid (C18:1 Δ 9) being predominant (13.878–17.673%).

Polyunsaturated fatty acids (PUFA) are the most abundant overall (43.235–48.510%), with arachidonic acid (C20:4 Δ 5, 8, 11, 14, 10.307–16.787%) as the major component, followed by eicosapentaenoic acid (EPA), linoleic acid (C18:2 Δ 9, 12, >9%), and docosahexaenoic acid (DHA, 6.407–8.871%).

These findings are generally consistent with literature reports [4] for crabs from the Gulf of Tunis, with no significant sex differences in PUFA content. Differences observed in stearic acid levels between males and females may reflect seasonal migration and dietary variations. MUFAs remain dominated by oleic acid, while major PUFAs include arachidonic acid, EPA, DHA, and linoleic acid. Variations in literature data may also arise from sample processing, such as cooking [29]. Studies from the southern Mediterranean CS [32] confirm that PUFA levels exceed SFA, with palmitic and stearic acids as the main SFAs, oleic acid as the main MUFA, and linoleic acid, arachidonic acid, EPA, and DHA as the principal PUFAs. Differences in PUFA concentrations are reflected in the ω6/ω3 ratios, which ranged from 1.126 to 1.672 (Table 3). These values, consistent with literature reports [4,32], remain within the optimal range and well below the maximum recommended daily intake of 4:1. The PUFA/SFA ratio ranged from 1.118 to 1.377, with the highest values observed in female appendages, aligning with previous studies where claw meat shows the highest ratios. All observed ratios are substantially above the minimum recommended value of 0.45 for a healthy diet.

The atherogenicity index (AI) and thrombogenicity index (TI) assess the balance of pro- and anti-atherogenic and thrombogenic fatty acids, respectively. In this study, AI ranged from 0.322 to 0.385 and TI from 0.375 to 0.407. Both values are below 1, suggesting a potential protective effect against cardiovascular diseases [4]. The hypocholesterolemic/hypercholesterolemic ratio (HH) ranged from 2.716 to 3.295 [4]. These values, substantially higher than those reported for chicken and rabbit meat, suggest potential beneficial effects on cholesterol metabolism [33,34]. Similarly, the health-promoting index (HPI), the reciprocal of AI, ranged from 2.601 to 3.138, markedly exceeding typical values for dairy products. Taken together, these values highlight a potential positive effect of CS on the human diet [35] while acknowledging that inter-study variability may arise from biological and environmental factors.

### 2.3. Quantification of Astaxanthin in CS Meats

Carotenoids are important antioxidant compounds contained in crustacean exoskeletons and in crustacean meats. Astaxanthin (AX; 3,3′-dihydroxy-β,β-carotene-4,4′-dione) is a major xanthophyll pigment in marine crustaceans and salmon, responsible for their coloration. In crustaceans like CS, AX is bound in carotenoproteins (e.g., crustacyanin), giving a blue-green hue that turns red-orange upon cooking due to protein denaturation [36,37,38]. It is concentrated in the exoskeleton and appendages, with reported levels in the CS exoskeleton ranging from ~30–98 µg/g, influenced by dietary and environmental factors. AX has applications in aquaculture, nutraceuticals, cosmetics, and food industries, owing to its pigmentation and antioxidant properties [39]. In edible crab tissues, studies report ~3–4 µg carotenoids/g of flesh, with AX constituting the majority (>36–67%) of total carotenoids [40].

As reported in Table 4, AX is quantified by HPLC-UV-MS. In this study, AX is detected in all samples, but reliable quantification is limited to claw meats. Notably, AX levels in claws differ significantly between sexes, with males containing substantially higher concentrations (67.1 µg/100 g FW) compared with females (13.58 µg/100 g FW). These findings have both biological and nutritional implications. Biologically, the sex-dependent and tissue-specific distribution of AX reflects differences in metabolic allocation, reproductive investment, or structural requirements of the exoskeleton [37,41]. Considerable variability among samples also reflects differences in specimen size or diet [41,42]. As previously reported, AX cannot be synthesised de novo and is accumulated through the diet [42]. From a nutritional perspective, claws represent a richer source of AX for human consumption, potentially offering higher antioxidant intake compared with breast meat [26,38,43]. This underscores the importance of considering both tissue type and sex when evaluating the nutritional value of crustacean products.

### 2.4. Analysis of Gastrointestinal Digestion Effects on Polyphenol Secondary Metabolites Composition, α-Glucosidase and ACE Inhibitory Activity

**Figure 2:**
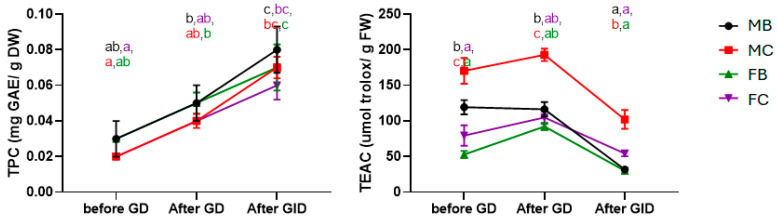
Total phenol content (TPC, (**left panel**)) and antioxidant activity (TEAC, (**right panel**)) before and after gastric digestion (GD) and gastrointestinal digestion (GID). Male body meat (MB), male claw meat (MC), female body meat (FB), female claw meat (FC). Values are expressed as mean ± SD. Different superscript letters indicate a significant difference among the means.

To verify the bioaccessibility of polyphenols and the total antioxidant activity, the samples from gastrointestinal digestion were analysed using common spectrophotometric methods [44]. The Folin–Ciocalteu assay results (Figure 2, left panel) demonstrate that the total phenolic content (TPC) significantly increased during gastrointestinal digestion when compared to pre-digestion content. Sex-related differences were not significant when considering the same time point (*p* < 0.05). The free radical scavenging activity shows an inverse trend compared to phenolic content (Figure 2, right panel). Antioxidant activity values (TEAC, Figure 2, right panel) remain comparable before and after gastric digestion (GD) but decrease significantly following simulated intestinal digestion (GID). This behaviour suggests that the antioxidant capacity of the CS is not solely attributable to phenolic compounds. The significantly higher activity observed in the claw meat of males, where astaxanthin concentration was also highest, suggests that this carotenoid, together with other lipophilic antioxidants, may contribute to the overall antioxidant activity. Similar trends have been documented in other crustaceans, where astaxanthin demonstrated potent radical scavenging activity in vitro and improved tissue antioxidant status in vivo [45,46].

The digested extracts were then analysed for their capacity to inhibit α-glucosidase and angiotensin-converting enzyme (ACE) at the concentration of 20 mg/mL, corresponding to the digested extract obtained from 100 mg of fresh crab meat, considering both its composition and digestibility. As reported in Table 5, all the tissues analysed inhibit both enzymes. α-Glucosidase is a key enzyme that catalyzes the final step of carbohydrate digestion by hydrolysing disaccharides and oligosaccharides into absorbable glucose. It is a crucial target for the development of antidiabetic medications since blocking its activity delays the intestinal absorption of glucose, lowering postprandial blood glucose rises. CS tissues were able to inhibit the enzyme, with inhibitory percentages (I%) ranging from 47.07 to 62.21%. The inhibitory activities are slightly higher for claw tissues with respect to breast. Similar results have been previously obtained with protein hydrolysates from green crab (*Carcinus maenas*); interestingly, the most active compounds were characterised by molecular weight < 3 kDa [47]. Similarly, *Procambarus clarkii* shell enzymatic hydrolysates showed inhibitory effects in vitro [48]. Regarding ACE inhibitory activity, all hydrolysed tissues show promising inhibitory percentages, with no differences among the groups. Similarly, recent research has shown that peptides produced from crustaceans can function as natural inhibitors of the ACE. For instance, Zhou et al. (2023) isolated the tripeptide ARL/I from the hydrolysate of *Marsupenaeus japonicus* shrimp heads endowed with a competitive inhibitory mechanism [49]. Other dipeptides with ACE inhibitory activity were identified in *Acetes chinensis* hydrolizates and in different crustacean processed waste [50,51]. These results emphasise the potential of crustacean-derived hydrolysates as nutraceuticals or functional food ingredients for the management of metabolic diseases. Although the inhibitory activity of the digested extracts is lower than that of drugs available on the market, the concentrations tested are compatible with those potentially present in the gastrointestinal tract after consumption of CS. Accordingly, these results indicate a potential inhibitory effect in vitro, which warrants further investigation in in vivo models.

## 3. Materials and Methods

### 3.1. Materials

The solvents and reagents used (analytical grade) were obtained from Sigma-Aldrich S.r.l. (Milan, Italy) and used without additional purification. Milli-Q quality water (Millipore, Milford, MA, USA) was used. *Callinectes sapidus* (CS) specimens were collected from the Ombrone River in September 2023 (Grosseto, Italy). Fifteen male and fifteen female specimens were selected, and the tissue was separated into carapace and appendage portions. Crab meat was subdivided into two anatomically defined fractions: appendage meat (C) and cephalothorax body meat (B). Appendage meat consisted of white muscle tissue manually extracted from the chelae and walking legs. Cephalothorax body meat consisted exclusively of the white muscle tissue located within the cephalothorax, following removal of the carapace. The brown meat (hepatopancreas) was carefully removed and discarded prior to sampling and was not included in any analyses. In female specimens, ovaries were visually inspected during dissection; when present, gonadal tissue was excluded from the cephalothorax meat fraction. Biometric data are reported in Supplementary Materials; yields were not optimised. The samples were provided by the MAGIAMARE-SIENA Research Group thanks to the FEAMP 2014–2020 project identification number 2/SSL/16TO2/RBC/21/TO.

The samples were weighed and then divided into six groups of five specimens each: three groups consisting of male crabs and three of female crabs. All samples were stored at −20 °C prior to analysis.

### 3.2. Analysis and Water, Fats, and Protein Content

One gram of each sample was used to calculate the water content. The material was weighed and dried at 105 °C for 14–16 h. To determine the water content, the initial weight was subtracted from the final weight. [52].

The nitrogen content of 1.0 g of dried material was determined using the Kjeldahl method. The crude protein content was calculated with a coefficient of 6.25.

The Folch method [53] was used for determining fat. Using an IKA Labortechnik T25 basic (IKA WERKE GmbH & Co., Staufen, Germany), CS (5 g) specimens were homogenised for five minutes before the extraction with CHCl_3_. The organic layer was filtered, washed twice with the “upper phase” (made from a CHCl_3_/CH_3_OH/H_2_O (53:27:20 *v*/*v*) mixture), and dried after being washed with 10 mL of KCl 0.7% solution. The extract’s fat content was determined by weighing it prior to FA analysis.

### 3.3. Gastrointestinal Digestion

Gastrointestinal digestion (GID) has been simulated in vitro as previously reported with some adjustments [54]. Using an IKA Labortechnik T25 basic (IKA Werke GmbH & Co., Staufen, Germany), 500 mg of dried CS specimens were homogenised for five minutes in five milliliters of TRIS/HCl buffer 50 mM pH 7.4. Following this, HCl 1M and pepsin (enzyme/substrate 1:250 *w*/*w*) were added to reduce the pH to 2. The mixture was incubated at 37 °C for two hours. The impact of gastric digestion (GD) has been assessed using the extract. After increasing the pH to 6.5 with NaOH 1M, trypsin and α-chymotrypsin (1:1) were added (enzyme/substrate 1:250 *w*/*w*). The mixture was incubated at 37 °C for two hours. After centrifuging the samples for 10 min at 5000 rpm, the supernatant was collected, filtered through 3 kDa filters (Amicon Ultra Millipore Sigma, Burlington, MA, USA), and stored at −20 °C until needed.

### 3.4. Determination of Amino Acids

Amino acids (AA) were quantified after gastrointestinal hydrolysis simulation. The Fmoc pre-derivatisation method and the previously described chromatographic conditions were used to quantify AA by HPLC. [54].

### 3.5. Quantification of Antioxidant Compounds and Antioxidant Activity

Total polyphenol content (TPC) has been assessed using the Folin–Ciocalteu procedure using gallic acid as a standard, as previously reported. The TPC was expressed in milligrams of gallic acid equivalent per gram of dry matter (mg GAE/g DW).

Radical scavenging activity was measured using the 1,1-diphenyl-2-picrylhydrazyl (DPPH) test, as published in [55], using Trolox as a reference compound. The radical scavenging activity was reported as TEAC (µmol of Trolox equivalent per gram of dry matter).

### 3.6. NMR Analysis

A total of 20 mg of lipophilic extracts were dissolved in 0.7 mL of CDCl_3_, which contained 0.03% (*v*/*v*) tetramethylsilane (Sigma Aldrich, Darmstadt, Germany). A Bruker Advance DPX 400 MHz spectrometer (Bruker Biospin, Rheinstetten, Germany) was used to record monodimensional ^1^HNMR at T = 298 K. A single pulse experiment using a 90° excitation pulse, four dummy scans, a three-second relaxation delay, and sixteen scans was used to obtain the ^1^H NMR of lipophilic extracts. The samples from GID hydrolysis were dissolved in 1.0 mL of 400 mM phosphate buffer/D_2_O (pH 7) with 3-(trimethylsilyl)propionic acid sodium salt (TSP) 1 mM as a standard. Samples were filtered and analysed using the Bruker zgpr sequence. The assignment of ^1^H NMR signals was performed by comparing them with the published data [52,56] and Chenomx NMR suite v 11 (Chenomx Inc., Edmonton, Alberta, Canada).

### 3.7. Determination of Fatty Acids

Fatty acids (10 mg) have been transesterified by reacting with 1 mL of BF_3_ methanolic solution, for 1 h at 100 °C [57]. As previously described in [57], the GC-FID studies were carried out utilising a split/spitless injector and a GC-FID (Perkin Elmer Clarus 500 GC, Waltham, MA, USA). The FA were quantified by considering the FA areas and their ratios for each resolved peak.

### 3.8. Quantification of Lipid Quality Indices

The indices associated with cardiovascular diseases, namely the index of thrombogenicity (IT), the atherogenicity index (AI), the hypocholesterolemic/hypercholesterolemic ratio (H/H), and the health-promoting index (HPI) have been calculated with the following formulas [58,59,60]:

(1) $$TI=\frac{C14:0+C16:0+C18:0}{0.5\times MUFA+0.5\times \omega 6+3\times \omega 3+\frac{\omega 3}{\omega 6}}$$

(2) $$AI=\frac{C12:0+4\times C14:0+C16:0}{PUFA\times \left(\omega 6+\omega 3\right)+MUFA}$$

(3) $$HH=\frac{cis-C18:1+\Sigma PUFA}{C12:0+C14:0+C16:0}$$

(4) $$HPI=\frac{\Sigma PUFA}{C12:0+4\times C14:0+C16:0}$$

### 3.9. Astaxanthin Quantification

Astaxanthin (AX) was quantified in the lipophilic extracts using HPLC-UV-MS. Chromatographic analyses were performed using an Agilent 1260 Infinity HPLC-UV-MS system connected to an Agilent MSD 6130 system (Agilent Technologies, Palo Alto, CA, USA). Spectra were collected in the scan range of m/z 100–1500. Chromatographic separation was carried out on a Phenomenex Kinetex C18 column (100 Å, 150 × 4.6 mm, 2.6 μm particle size) at a flow rate of 0.6 mL/min. Detection was performed at 480 nm, using a mobile phase consisting of Milli-Q water and acetonitrile (ACN) under the following gradient program (min/%A): 0/95, 1/95, 5/5, 19/5, 20/95. Quantification of astaxanthin was achieved by interpolation from a calibration curve. The calibration curve was constructed using the AX commercial standard (Merck, Darmstadt, Germany) over the concentration range of 0.00004–0.400 mg/mL, yielding a correlation coefficient (R^2^) greater than 0.99. The limit of detection (LOD) was 0.15 µg/mL, and limit of quantification (LOQ) was 0.46 µg/mL.

### 3.10. ACE Inhibitory Activity Assay

ACE inhibitory inhibition was determined on samples from gastrointestinal simulated hydrolysis [54]. The sample was mixed with 25 μL of ACE 0.1 mU/mL (rabbit lung ACE enzyme from Sigma-Aldrich, St. Louis, MI, USA), and 40 μL of HHL 6.5 mM and incubated for 30 min at 37 °C. A total of 85 μL of HCl 1 M was added to stop the enzyme process. Borate buffer was used in place of the sample solution to create a blank sample.

Hippuric acid was quantified by HPLC using an Agilent 1260 Infinity (Agilent Technologies, Palo Alto, CA, USA) and the previously described method [54] to identify ACE inhibitory activity. Separation was achieved using an LC-18 column (Phenomenex Ultracarb, Torrance, CA, USA, 4.6 × 150 mm, 5 μm) and an isocratic mixture of ACN (25%) and H_2_O containing 0.05% formic acid (75%) at a flow rate of 0.8 mL/min.

ACE inhibitory activity was determined using the equation:

(5) $$I\;(\%)\;=\left(1-\frac{Ainhibitor}{Ablank}\right)\times 100$$

*A_inhibitor_* is the peak area of HA, while *A_blank_* is the peak area of HA in the blank sample. The ACE inhibition was reported as inhibitory concentration percentages (I%).

### 3.11. α-Glucosidase Inhibitory Activity Assay

Amounts of 100 μL of α-glucosidase (1 U/mL) and 50 μL of digested CS sample (20 mg/mL) were pre-incubated at 25 °C for 10 min. Following the addition of 50 μL of the substrate p-NPG (5 mM p-nitrophenyl-α-D-glucopyranoside in phosphate buffer 0.1 M, pH 6.8), the samples were incubated for five minutes at 25 °C. A microplate reader was used to measure the absorbance at 405 nm.

### 3.12. Statistical Analysis

All of the data were analysed using GraphPad Prism 8.2 (GraphPad Software, La Jolla, CA, USA), and the findings were displayed as mean plus standard deviations (SD). The data’s homoscedasticity was verified by the Brown–Forsythe analysis. The Shapiro–Wilk test was applied to confirm the normality of the data. Nutrients, lipids, astaxanthin, and amino acids were examined using one-way analysis of variance (ANOVA). The means were compared by the Tukey post hoc test. Only when *p* was less than 0.05 were the data considered significant. Using different gastrointestinal phases of digestion and CS meats as fixed factors, two-way ANOVA was used to investigate the total polyphenol content and the antioxidant activity before, during, and after simulated digestion.

## 4. Conclusions

Native to the western Atlantic, the blue crab is an invasive alien species in Europe that affects natural habitats and causes important economic losses. According to the Blue economy principles, we evaluated the potential advantages of using CS as a food. We found that CS meat provides high-quality proteins with all essential amino acids and a lipid profile rich in PUFAs with low atherogenic and thrombogenic potential. Its abundance in antioxidant compounds, in particular astaxanthin, retained after simulated gastrointestinal digestion, suggested that CS could serve as a functional food. Interestingly, CS hydrolyzates have significant α-glucosidase and ACE inhibitory effects, suggesting important benefits for glycaemic control and cardiovascular health. This study provides novel insights by simultaneously evaluating the nutritional composition, bioactive compounds, and in vitro functional effects of CS meat, aspects not fully addressed in previous research. By combining proximate analysis with gastrointestinal digestion and enzyme inhibition assays, we advance understanding of the potential functional food applications of this species. While our study confirms the nutritional and bioactive potential of blue crab meat, its practical use is limited by the low meat yield and the costs associated with processing. These limitations should be considered when assessing the feasibility of valorising blue crab as a functional food, as they directly affect both economic and logistical viability. Nonetheless, our results provide valuable insights into the intrinsic nutritional and nutraceutical properties of the species, offering a basis for targeted applications despite these practical challenges. Overall, these findings position the blue crab as a promising functional food, offering both nutritional value and potential health benefits, while simultaneously supporting sustainable management of this invasive species.

## Figures and Tables

**Table 1:** Analysis of water, fats, and protein content in *Callinectes sapidus* *: percentage of water content, fats, and proteins on fresh weight (FW).

	Water (g/100 g FW)	Fats (g/100 g FW)	Proteins (g/100 g FW)
	Mean ± SD	Mean ± SD	Mean ± SD
MB	77.3 ± 0.53	0.65 ± 0.04 ^b^	18.51 ± 0.65
MC	76.91 ± 0.39	0.49 ± 0.02 ^a^	19.01 ± 0.68
FB	76.47 ± 0.75	0.64 ± 0.06 ^b^	19.41 ± 0.91
FC	76.72 ± 0.9	0.47 ± 0.06 ^a^	18.02 ± 0.48

* Results represent the mean ± SD of three experiments. Male body meat (MB), male claw meat (MC), female body meat (FB), female claw meat (FC). Values are expressed as g per 100 g of fresh weight. Different superscript letters indicate a significant difference among the means in each column, *p* < 0.05 by post hoc Tukey’s test. When no letters are shown for a given parameter, no statistically significant differences were found.

**Table 2:** Essential amino acid (EAA) composition of *Callinectes sapidus* reported in means ± SD (g/100 g FW) *.

AA	MB		MC		FB		FC	
	Mean	±SD	Mean	±SD	Mean	±SD	Mean	±SD
His	0.314	0.05	0.295	0.03	0.310	0.06	0.342	0.05
Thr	0.331	0.02 ^a^	0.195	0.03 ^b^	0.348	0.04 ^a^	0.275	0.04 ^a^
Val	0.277	0.01 ^a^	0.270	0.02 ^a^	0.115	0.04 ^b^	0.125	0.02 ^b^
Met + Cys	0.613	0.09	0.618	0.05	0.628	0.09	0.645	0.05
Lys	2.226	0.34	2.112	0.50	2.021	0.45	2.048	0.32
Ile	0.420	0.06	0.344	0.04	0.327	0.04	0.438	0.04
Leu	0.476	0.03	0.558	0.09	0.671	0.08	0.659	0.09
Phe + Tyr	0.171	0.05	0.160	0.03	0.197	0.04	0.187	0.06
Total EAA	4.827	0.87	4.559	0.79	4.617	0.84	4.718	0.64
Met	0.415	0.05 ^a^	0.426	0.02 ^a^	0.503	0.05 ^a^	0.524	0.02 ^b^
Cys	0.198	0.04	0.192	0.04	0.125	0.04	0.121	0.03
Phe	0.059	0.03	0.056	0.02	0.076	0.02	0.069	0.03
Tyr	0.112	0.02	0.104	0.01	0.121	0.02	0.118	0.03

* Results represent the mean ± SD of three experiments. Male body meat (MB), male claw meat (MC), female body meat (FB), female claw meat (FC). Different superscript letters indicate a significant difference among the means in each row, *p* < 0.05 by post hoc Tukey’s test. When no letters are shown for a given parameter, no statistically significant differences were found.

**Table 3:** Total FA composition of the CS expressed as mean % ± SD *.

	MB		MC		FB		FC	
	%	SD	%	SD	%	SD	%	SD
C12:0	1.234	0.561 ^a^	2.181	0.070 ^a^	3.502	0.273 ^b^	2.505	0.382 ^a^
C14:0	1.010	0.342	0.793	0.033	0.732	0.000	0.676	0.129
C15:0	0.596	0.055	0.612	0.058	0.494	0.080	0.601	0.068
C16:0	17.444	0.428	17.002	1.730	17.170	0.577	15.602	1.056
C17:0	1.819	0.192	1.811	0.235	1.894	0.041	1.855	0.077
C18:0	10.520	1.306 ^a^	12.257	1.826 ^ab^	14.182	0.145 ^b^	13.393	0.589 ^b^
C20:0	0.697	0.524	0.345	0.296	0.729	0.139	0.602	0.005
Σ SFA	33.720	0.434 ^a^	33.981	3.585 ^a^	38.703	0.978 ^b^	35.234	2.306 ^a^
C16:1 Δ 9	2.528	0.569	2.658	0.130	1.664	0.068	1.537	0.076
C16:1 Δ 11	1.064	0.171	1.103	0.172	0.664	0.080	0.664	0.017
C18:1 Δ 9	16.256	1.145 ^ab^	17.673	1.186 ^b^	14.793	0.122 ^a^	13.878	0.383 ^a^
C20:1 Δ 11	0.914	0.052	0.822	0.319	0.923	0.057	1.079	0.051
Σ MUFA	20.844	0.435 ^b^	21.492	0.163 ^b^	18.045	0.191 ^a^	17.403	0.353 ^a^
C16:2 (Δ 9.11)	0.578	0.014	0.837	0.089	0.480	0.004	0.439	0.020
C18:2 (Δ 9.12)	9.156	0.928	9.759	1.164	10.169	1.515	10.716	0.092
C18:3 (Δ 6.9.12)	1.959	0.090	1.867	0.261	1.596	0.245	1.844	0.111
C20:2 (Δ 11.14)	1.373	0.202	0.215	0.038	1.089	0.107	0.144	0.021
C20:4 (Δ 5.8.11.14)	15.619	1.871 ^bc^	14.532	0.629 ^b^	10.307	2.073 ^a^	16.787	1.090 ^c^
C20:5 (Δ 5.8.11.14.17)	10.295	0.363	10.730	0.726	10.742	0.392	11.272	0.638
C22:6 (Δ4.7.10.13.16.19)	7.795	0.813 ^ab^	7.421	0.324 ^ab^	8.871	1.144 ^b^	6.407	0.120 ^a^
Σ PUFA	45.435	0.001 ^b^	44.527	3.422 ^ab^	43.253	1.169 ^a^	48.510	1.298 ^c^
ω6/ω3	1.407	0.098 ^b^	1.416	0.012 ^b^	1.126	0.027 ^a^	1.672	0.017 ^c^
AI	0.354	0.036	0.339	0.005	0.385	0.020	0.322	0.042
TI	0.375	0.006	0.377	0.006	0.407	0.021	0.390	0.036
HH	3.095	0.247 ^a^	3.105	0.490 ^ab^	2.716	0.157 ^a^	3.295	0.394 ^b^
HPI	2.843	0.292	2.977	0.420	2.601	0.135	3.138	0.408

* Results represent the mean ± SD of three experiments. Male body meat (MB), male claw meat (MC), female body meat (FB), female claw meat (FC). Atherogenicity index (AI), thrombogenicity index (TI), hypocholesterolemic/hypercholesterolemic ratio (H/H), health-promoting index (HPI). Different superscript letters indicate a significant difference among the means in each row, *p* < 0.05 by post hoc Tukey’s test. When no letters are shown for a given parameter, no statistically significant differences were found.

**Table 4:** Quantification of Astaxanthin in CS. Data are expressed as mean ± SD.

	MB	MC	FB	FC
Astaxanthin (µg/100 g FW)	nq	67.1 ± 9.13 ^b^	nq	13.58 ± 4.02 ^a^

Results represent the mean ± SD of three experiments. Male body meat (MB), male claw meat (MC), female body meat (FB), female claw meat (FC). Different superscript letters indicate a significant difference among the means in each column, *p* < 0.05 by post hoc Tukey’s test. nq: not quantified; astaxanthin was below the limit of quantification.

**Table 5:** Analysis of α-glucosidase and angiotensin-converting enzyme (ACE) inhibitory activities after gastrointestinal digestion (GID) at the concentration of 20 mg/mL. Values are expressed as inhibitory percentages ± SD *.

	Inhibitory %	
	α-Glucosidase	ACE
MB	47.07 ± 2.31 ^a^	97.39 ± 0.98
MC	57.37 ± 3.16 ^b^	97.74 ± 1.25
FB	54.19 ± 2.05 ^b^	97.16 ± 1.91
FC	62.21 ± 2.13 ^c^	96.07 ± 1.56

* Results represent the mean ± SD of three experiments. Male breast meat (MB), male claw meat (MC), female breast meat (FB), female claw meat (FC). Different superscript letters indicate a significant difference among the means in each column, *p* < 0.05 by post hoc Tukey’s test. When no letters are shown for a given parameter, no statistically significant differences were found.

## Data Availability

The original contributions presented in this study are included in the article. Further inquiries can be directed to the corresponding authors.

## Supplementary Materials

The following supporting information can be downloaded at: https://www.mdpi.com/article/10.3390/ijms27010381/s1.
