# Supplementary material for: From Ecological Threat to Bioactive Resource: The Nutraceutical Components of Blue Crab (Callinectes sapidus)
Source: Int J Mol Sci. 2025 Dec 30;27(1):381. doi: 10.3390/ijms27010381 (PMC12786082; doi:10.3390/ijms27010381)
Supplement: Supplementary file 1 [file ijms-27-00381-s001.zip › ijms-4043261-supplementary.pdf]

From Ecological Threat to Bioactive Resource: The Nutraceutical Components of Blue Crab *(Callinectes sapidus)*

Annalaura Brai[1], Lorenzo Tiberio[1], Matteo Chiti[2], Federica Poggialini[1], Chiara Vagaggini[1], Guia Consales[2], Letizia Marsili[2] and Elena Dreassi[1*]

[1] Department of Biotechnology, Chemistry and Pharmacy, University of Siena, via A. Moro-53100 Siena, Italy1

[2] Department of Physical Science, Earth and Environment, Università degli Studi di Siena, Via P.A. Mattioli, 4, 53100 Siena, Italy

* Correspondence: elena.dreassi@unisi.it (ED)

Table S1. Biometric parameters of *Callinectes sapidus* exemplars analysed

Sample ID	Sex	Carapace length (cm)	Carapace width (cm)	Body weight (g)	Appendage meat (g)	Cephalothorax meat (g)	Total Yield
1CRB23	M	7.5	17.9	245	28.57	17.78	18.92
2CRB23	M	7	13.7	153	9.14	13.07	14.52
3CRB23	M	7.5	15	259	23.63	10.67	13.24
4CRB23	M	7.4	15	200	14.19	5.33	9.76
5CRB23	M	7.5	16.7	264	21.91	11.36	12.6
6CRB23	M	7.5	15	205	7.02	6.92	6.8
7CRB23	M	7.5	17.5	199	25.11	5.55	15.4
8CRB23	M	8.5	18	250	14.69	20.35	14.02
9CRB23	M	7.5	15.5	237	15.24	23.96	16.54
10CRB23	M	7	16	202	4.89	11.75	8.24
11CRB23	M	7.5	16.5	243	9.02	6.91	6.56
12CRB23	M	7	14.5	158	12.28	9.98	14.09
13CRB23	M	7.5	17.5	201	10.59	10.14	10.32
25CRB23	M	6.5	14.3	204	11.99	12.59	12.05
26CRB23	M	6.8	14.5	165	11.74	11.05	13.81
14CRB23	F	6.5	15.5	134	4.34	3.92	6.16
15CRB23	F	6.8	14.7	120	7.75	3.44	9.32
16CRB23	F	6.5	15	129	3.72	4.14	6.09
17CRB23	F	6.5	15	124	7.33	3.03	8.36
18CRB23	F	6.8	15.3	111	4.82	3.12	7.15
19CRB23	F	7.3	16	157	6.13	1.37	4.78
20CRB23	F	7.5	16	174	6.4	9.67	9.23
21CRB23	F	7.5	16.5	126	4.35	1.53	4.67
22CRB23	F	7.2	16.5	157	6.4	9.83	10.34
23CRB23	F	7	16.2	158	6.4	5.81	7.73
24CRB23	F	6.2	14	115	7.13	7.64	12.84
27CRB23	F	7.5	15.3	104	7.55	5.48	12.53
28CRB23	F	6.2	14.9	106	4.51	10.88	14.51

29CRB23	F	6.1	14	109	3.47	8.78	11.23
30CRB23	F	6.1	13.9	108	5.57	7.63	12.22
